# Environmental chemicals impact dog semen quality in vitro and may be associated with a temporal decline in sperm motility and increased cryptorchidism

**DOI:** 10.1038/srep31281

**Published:** 2016-08-09

**Authors:** Richard G. Lea, Andrew S. Byers, Rebecca N. Sumner, Stewart M. Rhind, Zulin Zhang, Sarah L. Freeman, Rachel Moxon, Holly M. Richardson, Martin Green, Jim Craigon, Gary C. W. England

**Affiliations:** 1 School of Veterinary Medicine and Science, University of Nottingham, UK; 2 School of Animal Rural and Environmental Sciences, Nottingham Trent University, UK; 3 Environmental and Biochemical Sciences, The James Hutton Institute, UK; 4 National Breeding Centre, Guide Dogs for the Blind Association, UK; 5 School of Biosciences, University of Nottingham, UK

## Abstract

Adverse temporal trends in human semen quality and cryptorchidism in infants have been associated with exposure to environmental chemicals (ECs) during development. Here we report that a population of breeding dogs exhibit a 26 year (1988–2014) decline in sperm quality and a concurrent increased incidence of cryptorchidism in male offspring (1995–2014). A decline in the number of males born relative to the number of females was also observed. ECs, including diethylhexyl phthalate (DEHP) and polychlorinated bisphenol 153 (PCB153), were detected in adult dog testes and commercial dog foods at concentrations reported to perturb reproductive function in other species. Testicular concentrations of DEHP and PCB153 perturbed sperm viability, motility and DNA integrity *in vitro* but did not affect LH stimulated testosterone secretion from adult testis explants. The direct effects of chemicals on sperm may therefore contribute to the decline in canine semen quality that parallels that reported in the human.

A significant decline in human semen quality over the last 70 years has been widely reported^1^,^2^. The first meta-analysis reporting this phenomenon included 61 studies selected over a 50 year period (1938–1991)^1^ and such a trend was confirmed by re-analysis with an additional 47 studies included^2^. More recently, a decline in semen concentration and morphology has been reported across the whole of France over a 17 year period^3^. Despite these reports, differences in laboratory methodologies and standards, sometimes included in the same meta-analyses, question the reliability of some of these studies^4^,^5^. Nevertheless, reports of declining sperm counts linked with epidemiological data on increased incidences of testicular cancer and genital tract abnormalities, is indicative of an adverse environmental effect on male reproduction^6^,^7^,^8^. Since these reproductive problems, termed by some as “testicular dysgenesis syndrome” (TDS), cluster in geographical areas^9^ they are thought to have a common aetiology and have been associated with endocrine perturbations in early life^10^. Exposure of developing males to environmental chemicals, particularly those with endocrine disrupting activity, is thought to be the initiator^11^. Although periods of development particularly sensitive to exposure encompass both pre-natal and pre-pubertal periods, environmental chemicals also perturb adult testis function e.g. gene expression and meiosis^12^. Although geographic divergences in exposure levels to oestrogenic or anti-androgenic compounds may explain the regional and national variations in reproductive health observed^13^,^14^,^15^, the data demonstrating such a relationship remains contentious^4^,^16^.

As ‘man’s best friend’ and closest animal companion, the dog shares the same environment, exhibits the same range of diseases, many with the same frequency, and responds in a similar way to therapeutic treatments. There is evidence that over the last 40 years, the incidence rate of canine testicular cancer has increased in parallel with changes seen in humans^17^,^18^. In addition, histological signs which characterise human TDS have recently been described in the dog^19^. These include seminiferous tubule abnormalities and testicular germ cell neoplasia *in situ* (GCNIS) cells which are known precursors of seminomas in the human^20^. Since human TDS includes a reduction in sperm count^21^ and an increased incidence of cryptorchidism, we hypothesised that the dog may exhibit similar manifestations of TDS and that this may be associated with exposure to endocrine disrupting chemicals. We therefore (1) monitored canine semen quality over a period of 26 years using standard and consistent techniques and (2) interrogated an extensive database of electronic health records for evidence of a temporal change in the incidence of cryptorchidism reported in male puppies. To explore a possible environmental aetiology, concentrations of chemical pollutants were measured in canine testes and semen collected from the same geographical area in which the temporal study had been carried out. The effects of chemicals, at testicular concentrations, were tested on endocrine and sperm function *in vitro*.

## Results

### Temporal changes in canine sperm motility, morphology and output

Sperm was collected from a population of stud dogs from a controlled breeding programme from 1988 to 2014. The coefficient of inbreeding for this population was low, with the annual mean coefficient for the 3 main breeds being: Labrador retriever = 0.05 (min 0, max 0.33); Golden retriever = 0.05 (min 0, max 0.34); German Shepherd Dog = 0.03 (min 0, max 0.28). The number of dogs within the programme each year varied between 42 and 97 throughout the period of study. Some dogs were examined during several consecutive years but the mean age of the population did not change each year because older dogs were retired and younger dogs were recruited to the breeding programme. Replacement dogs were selected based upon temperamental and health criteria but not fecundity.

**Figure 1:**
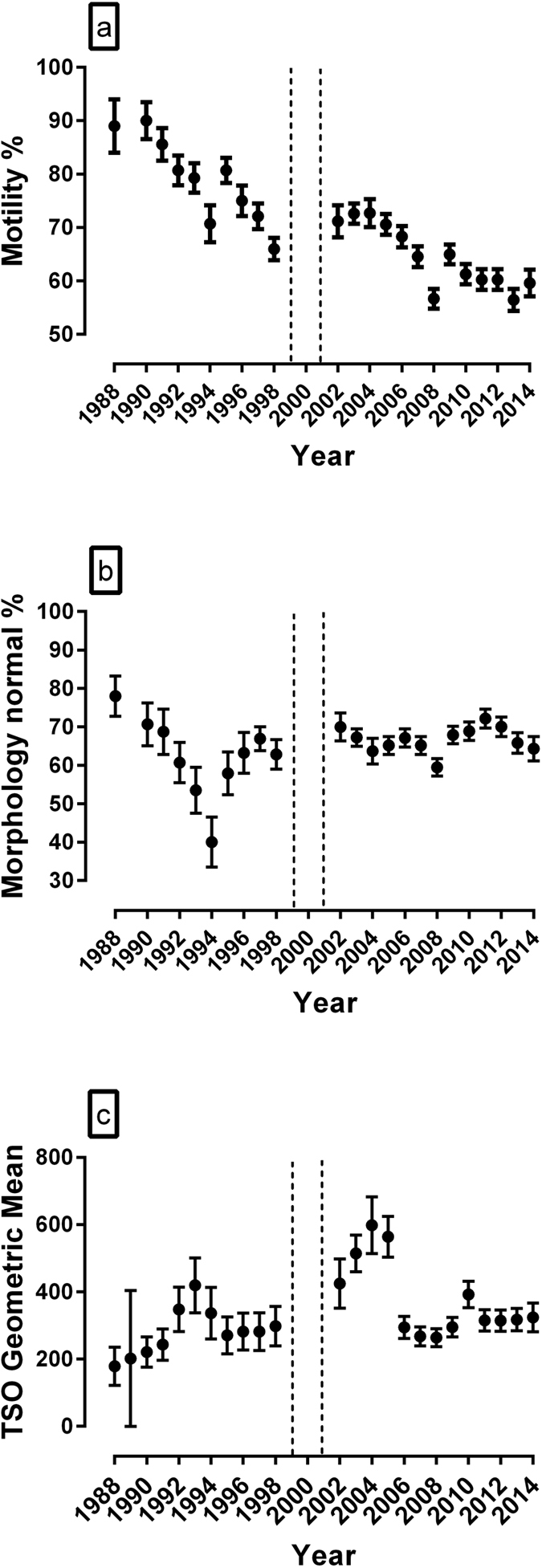
Temporal changes in semen quality in a population of stud dogs. Graphical summaries of the final statistical model parameters are shown for (**a**) Percentage Normal Motility, (**b**) Percentage Live Normal Sperm and (**c**) Total Sperm Output. In 1999 to 2001, dogs with poor semen quality were removed from the programme. Values of each parameter are given for years 1988–1998 and years 2002–2014 (n = 232 dogs: 42 to 97 in any one year). Statistical analysis: conventional multilevel models (MLwin version 2.22) used to evaluate change in semen quality over time accounting for repeated semen measurements. Error bars represent ± the standard error of each estimated mean point value from the model.

A decrease in the percentage of sperm with normal forward progressive movement (motility) was observed between years 1988–1998 (2.5% per year) with years 1992–1998 being significantly lower than 1988 (P < 0.05: Fig. 1a). After removal of dogs with the poorest semen quality between 1999 and 2001, there was a further decrease in motility (1.2% per year) with years 2007 to 2014 being significantly lower than 2002 (P < 0.05) (Fig. 1a). Between 1988 and 1994, the mean percentage of live normal morphology sperm declined (8.1% per year) with years 1992 to 1994 being significantly lower than 1988 (P < 0.01) (Fig. 1b). However, between 1994 and 1998, the percentage normal sperm increased and plateaued but still remained significantly lower than in 1988. After removal of dogs with the poorest semen quality between 1999 and 2001, there was no further significant decline with time (Fig. 1b). An increase in the total sperm output (TSO) was observed between years 1988–1998 with years 1992–1994 being significantly greater than 1988 (Fig. 1c). After removal of dogs with the poorest semen quality between 1999 and 2001, TSO increased with years 2004 and 2005 being significantly greater than 2002. This was followed by a decrease in TSO from 2005 to 2006 after which TSO remained unchanged through to 2014. During this period, years 2006–2009 and years 2011 to 2014 were significantly lower than 2002 (Fig. 1c). For all three parameters (percentage normal motility, percentage normal sperm and total sperm output), there were significant differences between breed and a non-linear decline with age of the dog.

Internal quality control procedures were applied to evaluate intra-technician variation as well as variation between the three technical staff who carried out the sperm analyses. The mean annual intra-class coefficient correlations for intra-technician variation were: Motility = 0.78 (95% confidence interval 0.34–0.94), Morphology = 0.62 (95% confidence interval, 0.05–0.88), Total sperm output = 0.85 (95% confidence interval 0.54–0.96). Three technical staff were involved in the semen analysis with change-over years of 1998 and 2008. Figure 1 shows a very slight decline in motility and morphology in 2008, however this does not appear to impact on the temporal trends for each of the 2 parameters. Indeed, the intra-class coefficient correlations for inter-technician variation were: Motility = 0.74 (95% confidence interval 0.45–0.88), Morphology = 0.38 (95% confidence interval, 0.13–0.78), Total sperm output = 0.74 (95% confidence interval 0.38–0.93).

### Temporal changes in the incidence of cryptorchidism and male/female ratio

**Figure 2:**
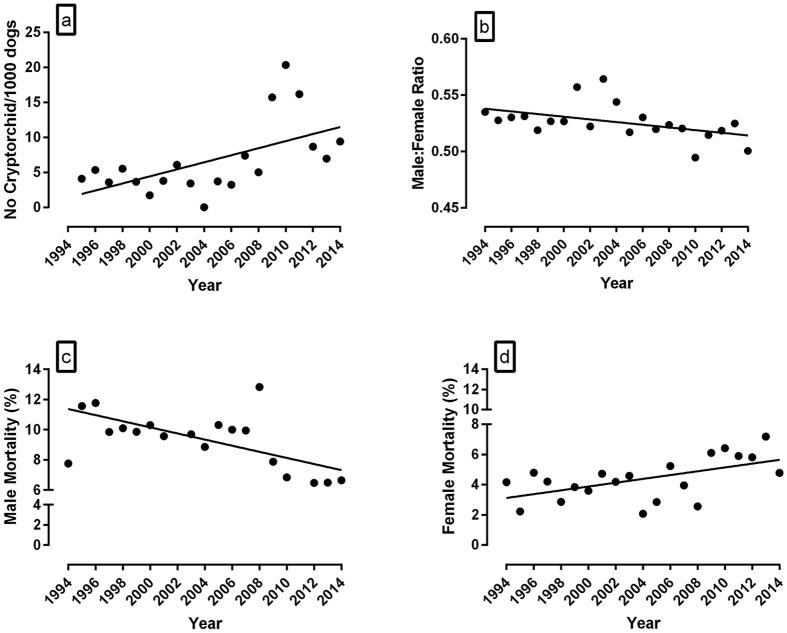
Temporal changes in the incidence of cryptorchidism, male:female ratio and mortality (stillbirth + early postnatal deaths) in puppies generated at the National Breeding Centre from 1994/5 to 2014 (**a**) Incidence rate of cryptorchidism (R^2^ = 0.33, P = 0.008, n = 20). (**b**) Male:female ratio (No.Males/No.offspring per litter) of all pups born (including stillbirths and early postnatal deaths) (R^2^ = 0.20, P = 0.04, n = 20). (**c**) Male mortality (R^2^ = 0.32, P = 0.008, n = 20). (**d**) Female mortality (R^2^ = 0.31, P = 0.009, n = 20). Statistical significance was assessed by linear regression (GraphPad Prism). Mean number of male dogs born per year (alive + still born) = 651.25 ± 14.79.

The incidence rate of cryptorchidism in puppies from 1995 to 2014 was assessed from records maintained on the National Breeding Centre database (average number of male dogs per year = 651.25 ± 14.79). Figure 2a demonstrates a significant increase in cryptorchidism from 1995–2014 (R^2^ = 0.33, P = 0.008, n = 20). Over the same period of time, there was a decrease in the proportion of total males born per litter (Fig. 2b: R^2^ = 0.20, P = 0.04, n = 20, male:female ratio = number of males/total pup number per litter). However the decline was lost when stillbirths and post-natal mortalities were excluded. Further analysis of fetal and neonatal deaths revealed both a decline in male mortality (Fig. 2c: R^2^ = 0.32, P = 0.008, n = 20) and an increase in female mortality (Fig. 2d: R^2^ = 0.31, P = 0.009, n = 20).

### Establishing concentrations of environmental chemicals in canine adult testis and semen

**Figure 3:**
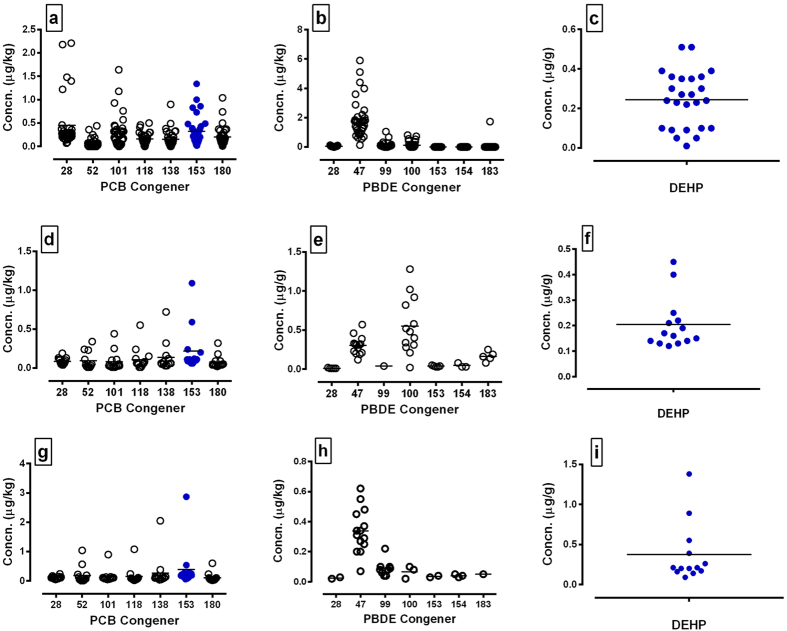
Concentrations of environmental chemicals in adult dog testes and commercially available dog food. (**a**,**d**,**g**) PCB congeners, (**b**,**e**,**h**) PBDE congeners and (**c**,**f**,**i**) DEHP were measured in (**a**–**c**) testes, (**d**–**f**) dry dog food and (**g**–**i**) canned dog food. Each circle represents a single testis or food sample. Blue circles show PCB 153 and DEHP that were detected above base levels in testes and food and selected for *in vitro* studies. Line indicates mean value.

Canine testes were subjected to EC analysis to establish (1) presence in the tissue (2) if concentrations are compatible with those associated with infertility in the human and (3) to determine canine testicular concentrations for *in vitro* functional or viability studies with testis and sperm. Seven polychlorinated bisphenol (PCB) congeners, 5 polybrominated diphenyl ether (PBDE) congeners and diethylhexyl phthalate (DEHP) were detected in testis (Fig. 3a–c).

Sperm rich fractions (fraction 2) of 14 canine ejaculates were collected and two pools generated of 5 samples and one of 4 samples. This provided enough cellular material for chemical analysis of PCB and PBDE congeners (but not DEHP). All 3 pools had detectable levels of 7 PCB congeners including PCB 153 (13.05 ± 3.18) as well as PBDE 28 and PBDE 47. Two of the pools also had detectable levels of PBDE 99 and PBDE 100 (Supplementary Figure S1a,b).

Since PCB153 and DEHP were detected in a majority of testis samples (Fig. 3a–c) and since PCB153 was also detectable in pooled ejaculate (Supplementary Figure S1a), these chemicals were selected at testis levels for *in vitro* studies using canine testis explants and sperm cultures. Mean testis levels for PCB153 and DEHP were calculated as 0.063 ± 0.01 ng/g (n = 34) and 0.182 ± 0.044 ug/g respectively (n = 25).

### Establishing concentrations of environmental chemicals in commercial dog foods

To investigate a possible nutritional source of ECs, a range of commercially available dog foods were subjected to the same array of analyses as the testis and ejaculate samples. Consistent with testis and ejaculate measurements, seven PCB congeners, 5 PBDE congeners and DEHP were detected in dry (Fig. 3d–f) and wet (Fig. 3g–i) dog food respectively. Amongst the dry dog food samples, one sample designed for puppies (1 to 24 months of age) had higher concentrations of 4 PCB congeners (101, 118, 138, 153) and PBDE 47 relative to the other samples tested. One popular wet dog food had higher concentrations of PBDE 47 and all 7 PCB congeners relative to the other food brands tested. DEHP and some PCB and PBDE congeners predominant in some of the dry and wet foods were also predominant in a majority of testis samples. This included PCB153 and DEHP selected for *in vitro* studies.

### PCB153 and DEHP induce contrasting effects on parameters of sperm movement measured by Computer Assisted Sperm Analysis (CASA)

**Figure 4:**
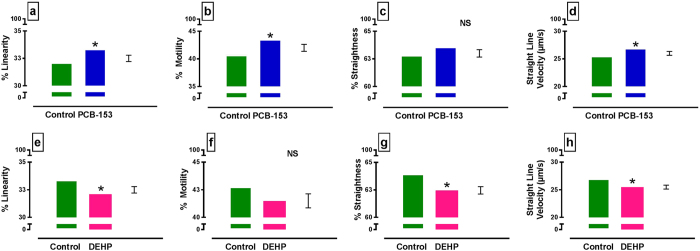
Computer Assisted Semen Analysis (CASA) of canine sperm exposed to testicular concentrations of PCB153 and DEHP. (**a**–**d**) Effects of PCB153 and (**e**–**h**) DEHP are shown for (**a**,**e**) percent sperm linearity, (**b**,**f**) percent sperm motility, (**c**,**g**) percent straightness and (**d**,**h**) straight line velocity. Overall chemical effects are shown (n = 7 dogs). Note: chemicals were tested at 2x, 10x, 100x mean testis concentration [mean testis levels for PCB153 0.063 ± 0.01 ng/g (n = 34) & DEHP: 0.182 ± 0.044 ug/g (n = 25)]. There was no interaction with concentration or time. Predicted values from general ANOVA model are presented. *P < 0.05. NS = not significant. Error bar = SED.

By CASA analysis (Hobsons sperm tracker), PCB153 accentuated percent linearity (P < 0.05), percent motility (P < 0.05) and straight line velocity (P = 0.05) (Fig. 4a,b,d). In contrast, DEHP reduced each measure of sperm movement reaching significance in percent linearity (P < 0.05), percent straightness (P < 0.05) and straight line velocity (P < 0.05) (Fig. 4e,g,h) but not in percent motility (Fig. 4f).

### PCB153 and DEHP perturb sperm vitality and DNA fragmentation *in vitro* but do not affect testosterone secretion from testis explants

**Figure 5:**
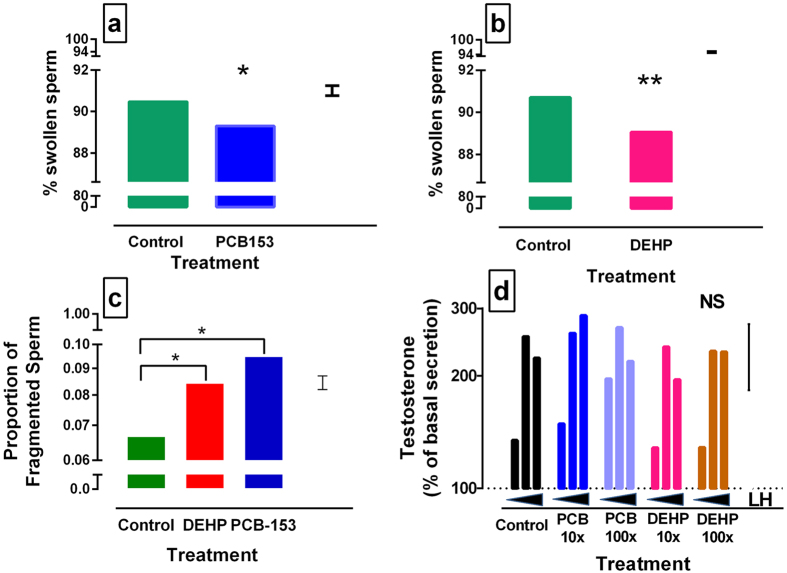
Effects of testicular concentrations of PCB 153 and DEHP on sperm vitality, DNA fragmentation and testosterone secretion from testis explants. (**a**,**b**) Sperm vitality was measured by the hypo-osmotic swelling test following incubation with PCB 153 (**a**) and DEHP (**b**) [n = 7 dogs]. (**c**) Sperm DNA fragmentation was measured by sperm chromatin dispersion assay (n = 10 dogs). (**d**) LH stimulated testosterone secretion by canine testis explants was measured in the presence of each chemical independently. Dotted line (100%) = explant + media only (no LH, no chemical). Chemicals were tested at 2x, 10x and 100x (**a**–**c**) or 10x and 100x (**d**) the concentration detected in testis [mean testis levels for PCB153 0.063 ± 0.01 ng/g (n = 34) & DEHP: 0.182 ± 0.044 ug/g (n = 25)]. (**a**–**d**): Predicted values from general ANOVA model are presented. *P < 0.05, **P < 0.01. NS = not significant. Error bar = SED.

Canine sperm, collected immediately after ejaculation, was incubated with PCB153 or DEHP at 2x, 10x and 100x testicular concentrations. Sperm vitality was measured at 0 and 4 hours and DNA fragmentation at 0, 2 and 4 hours. Sperm vitality, measured by the hypo-osmotic swelling test, was significantly reduced by PCB153 (P < 0.05) (Fig. 5a) and DEHP (P < 0.01) (Fig. 5b). Sperm DNA fragmentation was increased following chemical exposure (overall treatment effect: P < 0.001, PCB153 and DEHP considered independently: P = 0.05) (Fig. 5c). There was no evidence of a difference with concentration or an effect of time.

Adult canine testes were maintained *in vitro* for 24 hours and normal endocrine function of the explants was demonstrated by a dose-dependent increase in testosterone in response to the addition of LH (P < 0.01). The addition of PCB153 and DEHP at 10x and 100x mean testis concentrations, to the explant cultures exhibited no inhibitory effects on LH-induced testosterone secretion (Fig. 5d).

## Discussion

The findings from the current study are significant for the following reasons. They document, for the first time, a decline in the semen quality of a population of stud dogs over a period of 26 years. Although in some years semen quality parameters temporarily increased as noted in other studies^22^, there was a repeated decline in motility even after removal of animals with the poorest semen quality. Contemporaneous with the decline in semen quality, offspring from the same population of dogs showed an increased incidence of cryptorchidism from 1995 (first available records) to 2014, a decline in the proportion of male pups at birth, and a temporal increase in female mortality. The decline in number of males relative to the number of females was lost when stillborn pups/early post-natal deaths were excluded. Significantly, we report that the endocrine disrupting chemicals PCB153 and DEHP are detectable in canine testes collected from dogs from the same location as the temporal study. Furthermore, the same chemicals were detected in a range of commercially available dog foods. Since testicular concentrations of these chemicals directly perturbed sperm motility and viability, this may be a mechanism by which environmental chemicals directly affect male fertility.

In the current study, it was not our intension to demonstrate temporal changes in semen or testicular chemical content and to correlate with changes in semen quality. Indeed, such screening is confounded by the need to pool individual semen samples, the lack of historical samples and access to the technology at the start of the study. Rather, we have focussed on temporal changes in semen quality and have demonstrated that ECs are able to influence motility and other parameters of semen quality. Similarly, correlating the decline in semen quality in individual stud dogs with cryptorchidism in their offspring would be difficult since an individual stud dog may have a breeding career of 8 years and may produce several hundred male offspring. Thus although the relationship between semen quality, cryptorchidism and ECs is not demonstrated through a longitudinal or cross-sectional study design, we postulate that the rapidity of the temporal changes observed are indicative of an environmental aetiology and that our *in vitro* data support this contention.

The original meta-analysis reporting a decline in human semen quality spanned a period of 53 years (1938–1991)^1^. Although this time frame is extensive, the study outcomes have recently been criticised on the basis of changes in laboratory methods, training of laboratory personnel and improved quality assurance over the years^4^. Since additional studies have either supported or refuted the original claims, declining human semen quality remains controversial. In contrast, data reported here on canine sperm quality have been generated using sperm analysis methods that have remained consistent throughout the 26 years of the study with uniform input from three experienced technical staff and one of the senior authors (GE). Indeed, although there was some variability between technical staff, this was in line with, or better than, other studies^23^. Given that the current study is devoid of the confounding factors inherent in human meta-analyses, we present a unique dataset showing a reliable temporal decline in semen quality which, in this case, was observed in a controlled population of stud dogs. This raises the tantalising prospect that the decline in canine semen quality has an environmental cause. Indeed it is unlikely that the effect is heritable since in this population of dogs heritability measures were low for all parameters except for the heritability of high sperm motility and low total sperm output^24^.

The decline in percent normal morphology sperm from 1988 to 1994 followed by an increase and apparent subsequent plateau is difficult to interpret. Although a temporal decline in human sperm morphology has been reported and attributed to environmental factors^3^, the degree to which this accounts for the temporal trends in canine sperm morphology is uncertain. Furthermore, the increase in total sperm output contrasts with the decline in motility and morphology and with human studies and meta-analyses showing a decline in sperm counts. One factor that may have influenced the increased sperm output from 2002 to 2005 is that between 1999 and 2001 dogs with poor semen quality were removed. Since this included dogs with less than 150 million sperm, their removal may have constituted a bias towards increased sperm output. Indeed a similar temporal increase in sperm output has been reported in bull semen between 1985 and 1995, and a comparable selection bias was suggested^22^.

In the human, cryptorchidism affects 2 to 9% of new born infants^25^ and published data generally supports a temporal increased incidence and clear geographic differences in the prevalence^25^,^26^. As a key manifestation of “testicular dysgenesis syndrome” in the human, data presented here illustrates a similar increased incidence of cryptorchidism in a population of stud dogs comprising 5 breeds. While inbreeding in dogs has been linked to cryptorchidism, it is generally accepted that the pathology is underpinned by an interaction between genetic, epigenetic and environmental factors^27^. It is however difficult to tease out the relative contributions of these drivers. Whilst we were unable to undertake chemical analysis on the cryptorchid testes, it is an interesting observation that all of these cryptorchid pups were produced from the stud dogs donating the semen and described in Fig. 1. Clearly, study of the effect of exposure of the pregnant dam on male fetuses are warranted. In a study of 1,339 litters of 4 different canine breeds, litters with 1 or 2 cryptorchids were reported to exhibit an imbalanced sex ratio in favour of males^28^. In a separate canine study in which cryptorchidism carriers were intentionally selected for further breeding, a similar increased male to female ratio (in favour of males) was observed at weaning along with reduced numbers of females per litter^29^. Both of these reports contrast with the current study in which cryptorchidism carriers were purposely excluded from the breeding stock and there was no effect on the male:female ratio of live pups at birth. However, if stillbirths and early post-natal deaths were included, there was a decrease in male:female ratio, at the expense of males, accompanied by an increase in female mortality and decline in male mortality. Although previous studies of temporal trends in canine stillbirth rates have shown a significant fluctuation with time (1978–2005), gender specific changes have not previously been described^28^.

In the current study, although the contemporaneous increase in cryptorchidism and female mortality likely occurs through a separate mechanism, a lethal effect on females induced by genes that cause cryptorchidism has previously been proposed^30^. However, this contention was put forward to account for a ‘high sex ratio’ in favour of males in litters with cryptorchids, which contrasts with the reduced proportion of males per litter reported in the current study. Notwithstanding, the rise in cryptorchidism, fall in male:female ratio and increased female mortality all alter with time and the former is also related to declining sperm quality for which there is substantial evidence of an environmental trigger.

The dog is probably man’s closest companion and by sharing the same habitat, is likely to be exposed to similar environmental conditions including environmental chemicals. This was especially true in the current study since the dogs lived in homes with their handlers. Twelve chemicals detected in adult dog testes (DEHP, 7 PCB congeners, 4 PBDE congeners) were also detected in the 15 commercially available dog foods analysed. Since this indicated a possible food source of exposure, two chemicals (PCB153 and DEHP) were selected for further *in vitro* study on the basis of (1) high abundance in both testis and some pet foods and (2) both chemicals, or their metabolites, are reported to be present in seminal plasma of farm animals and in men at similar concentrations to those reported in the current study^31^,^32^. In addition, dietary/oral exposure to DEHP or PCB153 alters semen quality in animal models^33^,^34^ and human male infertility has been associated with elevated levels of a mix of PCB congeners in seminal plasma and DEHP^35^. In the current study, 6 PCB congeners and 4 PBDE congeners were detected in entire ejaculate pools at concentrations greater than that observed in the testes. Since testicular concentrations of PCB 153 and DEHP directly impacted on sperm function and viability, it is axiomatic that the higher concentrations found in seminal plasma will similarly alter the parameters measured.

In the current study, we recognise that the lowest chemical concentration used did not equate with a “no adverse effect level (NOAEL)”. Although a wider range would be preferable, we have shown effects of PCB153 and DEHP at low environmentally relevant concentrations. The mechanism by which PCB153 and DEHP alter sperm motility and viability is uncertain. Indeed, previous studies of PCB and DEHP effects on sperm quality have yielded mixed results. For example, in the human, a number of studies report an inverse relationship between serum PCBs and sperm quality^36^. In separate studies of seminal ECs across human populations selected on the basis of variable organochlorine exposure, increased seminal PCB153 was consistently associated with reduced sperm motility^37^,^38^. In contrast, the sum of 4 PCB congeners in human blood plasma, including PCB153, has been reported to be lower in samples from men with infertility and, although negatively associated with testosterone, was positively associated with sperm quality^39^. Although this latter study is consistent with the positive effect we report of PCB153 on sperm motility, establishing a clear effect of single or multiple PCB congeners is complex, likely due to effects of confounding blood and seminal ECs. Similar mixed results have been observed in relation to DEHP and sperm quality. For example, one study from China reported that human urinary phthalate metabolites, measured as an index of DEHP exposure, were associated with increased sperm DNA damage and apoptosis^40^. In contrast, a separate study from the USA found very little association between DEHP metabolites and a range of sperm parameters including motility^41^. A further study from Sweden however, reported a negative correlation between urinary DEHP metabolites and sperm motility^42^. Phthalate esters have also been measured directly in human semen and reported to be inversely associated with sperm motility^43^. Notably, the authors report that the same inverse relationship was recapitulated *in vitro* when semen concentrations of phthalates were added directly to sperm. These findings support our *in vitro* findings of an inhibitory effect of DEHP on parameters of sperm motility.

In the current study, although the chemical effect on any independent parameter of sperm motility was subtle, the consistent differing effects of PCB153 and DEHP on all four motility parameters may have greater physiological relevance. Both chemicals are known endocrine disruptors with PBDE and PCB considered to have pro-estrogenic or anti-androgenic activity^44^ whereas DEHP and its primary metabolite mono-2-ethylhexyl phthalate (MEHP) are reported to exhibit pro- and anti-androgenic activity respectively^45^,^46^. The differing effects that PCB153 and DEHP have on sperm motility may reflect their respective actions on estrogen or androgen receptors, both of which are expressed by sperm^47^,^48^. However, it is recognised that many environmental chemicals operate through other endocrine and non-endocrine mechanisms, one or more of which may account for the direct effects on reproductive function. In support of this, neither chemical blunted LH stimulated testosterone secretion however acute direct effects were observed on sperm motility. In the human, environmental concentrations of DEHP and PCB153 have previously been reported to reduce sperm motility^49^,^50^ and interactions have been reported for some PCBs and phthalates mediated by PCB metabolites and enzymes important for phthalate metabolism^51^. With respect to DEHP exposure *in vivo*, the main anti-androgenic effects reported are attributed to its primary metabolite MEHP^52^. For example the exposure of canine testis interstitial cell suspension cultures to MEHP both inhibits hCG induced testosterone secretion and increases basal testosterone secretion^53^. In the current study, although DEHP adversely affected motility, vitality and DNA fragmentation, it had no anti- or pro-androgenic effect on testosterone secretion. This likely reflects the use of DEHP rather than MEHP and/or the lack of metabolic breakdown in culture, as reported for cultured rat fetal testis cells^46^ or relatively low levels of activity as reported in human testis extract^54^. The lack of a pro-androgenic response may reflect the use of canine adult testis explants rather than fetal or adult dispersed cells as described above^46^,^53^. Although we recognise that expanding our study to encompass the effects of PCB and DEHP metabolites on sperm would help consolidate the mechanism, our data are indicative of an interaction between PCB153 and DEHP as reported by others. Furthermore, our observation of an increase in sperm DNA fragmentation and reduced viability, as measured by the hypo-osmotic swelling test, supports previous studies on human sperm showing similar effects and also, increased urinary phthalate concentrations are reported to be associated with lower sperm counts and altered morphology^55^,^56^. Notwithstanding, it is recognised that the response of sperm to chemicals *in vivo* will inevitably reflect exposure to a more complex mixture of pollutants, many of which may interact. Thus establishing clinical relevance and ‘cause and effect’ would require the generation of a mixture of chemicals representative of what is present in semen and/or testis.

In conclusion, this study demonstrates that in a population of stud dogs, sperm motility has declined over a 26 year period. Although the mechanism remains to be determined, we have shown that chemicals present in testis and ejaculate directly affect sperm function and viability. Since the increased incidence of cryptorchidism coupled with declining sperm quality in males is indicative of canine “testicular dysgenesis syndrome”, the domestic dog may be a useful sentinel for the study of environmental influences on human male fertility.

## Methods

### Animals and sample collection

All sperm and testis collections and measurements were performed in accordance with relevant guidelines and regulations and all experimental protocols were approved by the University of Nottingham’s Animal Welfare and Ethical Review Body. All samples were collected as part of routine breeding management. Sperm was collected as part of routine reproductive examination of stud dogs and residual canine testis was collected from normal healthy dogs (n = 35) during routine castrations and subject to full owner consent. Testis samples were stored at −20 °C until testing.

### Monitoring of canine sperm motility, morphology and total sperm output (1998–2014)

Sperm was collected annually from stud dogs used in an assistance dog breeding programme. The dogs were of five breed types; Labrador retriever, golden retriever, curly coat retriever, border collie and German shepherd. All dogs lived with their handler and visited the breeding centre for the purpose of breeding or temporary boarding. The number of dogs within the programme varied each year throughout the period of study (n = 42 to 97) and data were available on a total of 1925 ejaculates from 232 different dogs. All semen was collected and evaluated by the last author (GCWE) or one of three technicians trained by him. Collections were performed in the absence of a teaser bitch by digital manipulation of the penis as previously described^57^. The percentage of sperm with normal forward progressive motility (Percentage Normal Motility:PNM), the percentage of morphologically normal live spermatozoa (Percentage Normal Sperm:PNS) and the total sperm output (TSO) was measured using standard techniques as consistently outlined in the 1987, 1992 and 2010 WHO reference manuals and previously applied to canine semen^58^,^59^,^60^,^61^. Morphology slide smears were prepared by mixing a sample of sperm rich ejaculate with nigrosin/eosin at a ratio of 1:4 (ejaculate to stain) for 2 minutes. A histological smear of the mix was examined using a phase contrast microscope at 640 X magnification (oil emersion)^60^,^62^. Some of the dogs were examined on several consecutive years of the study, commencing when they entered the breeding programme until they left the breeding programme.

In 1998, it was clear that the PNM for the population was significantly lower than it had been in the majority of the 10 preceding years. Therefore in years 1999 to 2001, dogs with poor semen quality (dogs with any of the following measurements: <60% normal forward progressive motility, <60% morphologically normal sperm, <150 million sperm) were removed from the programme. This resulted in an overall improvement in the semen quality of dogs in the programme, especially TSO. On the basis of this intervention, trends in semen quality were evaluated and data are presented as two separate periods from 1988–1998 and 2001–2014 inclusive.

### Sperm collection for measurement of and exposure to environmental chemicals

In order to measure chemicals in canine sperm, ejaculate was collected from 14 different stud dogs from the same population in which the decline in semen quality was identified. The sperm rich fraction of each (fraction 2) was collected and two pools of 5 samples and one of 4 samples generated for analysis.

To test the effects of environmental chemicals on parameters of sperm function and viability, ejaculates were collected from the same population of breeding dogs but from a separate cohort of seven normal healthy stud dogs aged between 2 and 8 years. An abstinence period of a minimum of one week was observed and all ejaculates were subjected to the routine fertility assessment previously described: motility, volume (ml), sperm concentration, total sperm output and total number live sperm.

### Chemical measurements

Adult dog testes (n = 35), canine ejaculate (n = 14) and a range of commercially available dog foods (dry dog biscuit: n = 13; canned wet meat: n = 12) were tested in an ISO17025 accredited laboratory for PCB congeners (28, 52, 101, 118, 138, 153, 180), PBDE congeners (28, 47, 99, 100, 153, 154, 183) and DEHP. The analytical methods used to determine concentrations used standardised extraction protocols and gas chromatography (GC) analysis as previously described^63^,^64^. Mean testicular concentrations (MTC) were calculated in a cohort of testes (n = 34: PCB, n = 25: DEHP) as a determination of background tissue concentrations which equate to environmental levels.

### Preparation of sperm and chemicals for *in vitro* analyses

PCB153 and DEHP were tested individually or in combination at a final concentration of 2X, 10X and 100X mean testis concentration. Chemicals were dissolved in either 100% ethanol or DMSO and diluted to 0.02% in Phosphate Buffered Saline (PBS) [sperm motility and vitality studies: DEHP 0.2% ethanol]^65^. Each sperm sample was diluted (20:1) in PBS and equal volumes of sperm and chemical solutions were mixed and incubated for subsequent analyses. Control sperm cultures for motility, HOS (n = 4) and DNA fragmentation (n = 10) were incubated in PBS with 0.1% solvent only (working concentration).

### DNA fragmentation (sperm chromatin dispersion assay)

Diluted semen samples were cultured for 0, 2 and 4 hours with concentrations of DEHP or PCB-153 equivalent to 2x, 10x and 100x those measured in testes. Each semen sample was individually combined with an equal volume of 1% low-melting point agarose. A 15 μl aliquot of the sperm-agarose suspension was pipetted onto a polysine slide (Thermo Fisher Scientific, Loughborough, UK), pre-coated with a 1% agarose solution, and after application of a coverslip, it was left to solidify at 4 °C for 5 minutes. Following coverslip removal, slides were immersed in acid denaturing solution (0.08 M HCl) for 7 minutes. Slides were then incubated with lysing reagent (0.8 M DTT, 0.4 M Tris, 2 M NaCl, 1% triton-X) for 20 minutes. Lysing of cells was stopped by immersion in dH2O for 5 minutes prior to a series of sequential ethanol dehydration steps (70%, 95% and 100%). Visualisation occurred via submersion of slides in Diff-Quik (Eosin-Y) followed by Methylene Blue for 7 minutes and analysed via brightfield microscopy at 640X magnification (oil emersion). A minimum of 200 sperm were counted and percent fragmentation calculated. Sperm not showing the characteristic halo, indicative of chromatin dispersal, were considered fragmented.

### Hypo-osmotic swelling test (HOST)

As previously described^66^, a hypotonic solution (150 mOsm/l1^−1^) was prepared using fructose (Sigma, Poole, UK), sodium citrate (Sigma, UK) and reverse osmosis water. The sperm rich portion of the ejaculate (0.1 ml) was incubated in 1.0 ml of hypo osmotic solution for 30 minutes at 37 °C. After mixing, 10 ul was smeared onto a glass slide and air dried. Two hundred sperm were observed with a phase contrast microscope at 400X magnification. The assay was carried out on all samples at 0 and 4 hours and the percentage of cells showing osmotic stress calculated.

### Computer Assisted Sperm Analysis (CASA)

Semen (sperm rich fraction), mixed with chemical culture media, was incubated in a water bath at 37 °C. Ten μl of mixed semen was placed on a Helber counting chamber slide (depth 0.02 mm: Hawksley, UK), covered with a 22 × 22 mm coverslip and assessed for motility using a Hobson Sperm Tracker (Hobson Tracking Systems Ltd, Sheffield, UK). Standard settings recommended by the supplier were used and each treatment tracked for one minute at four time points (0, 1, 2, 4 hours) as previously described^67^. All CASA parameters were recorded for each chemical (PCB 153, DEHP and mix PCB153/DEHP) at each concentration (x2, x10, x100 mean testis concentration).

Fourteen parameters of sperm motility were measured using the CASA system. These were progressive motility (WHO laboratory manual)^60^, straight line velocity (VSL), curvilinear velocity (VCL), average path velocity (VAP), straightness of track (STR), linearity of track (LIN), amplitude of lateral head displacement (ALH), beat cross frequency (BCF), dance mean (DMN), pause variation of VSL (PFT), Mean angular displacement (MAP), percent active, percent hyperactive and percent motile^60^,^68^,^69^.

### Canine adult testis explant culture and testosterone ELISA

Left and right testes were collected from routine castrations and immediately transferred into pre-warmed serum free Dulbeco’s Modifided Eagle Medium (DMEM: Sigma-Aldridge Company Ltd) supplemented with high fructose, penstrep (100 ug/ml) and gentamycin (50 ug/ml). After removal of the tunica vaginalis and epididymis, the testis was bisected and a 2.5–3 mm disk (cross section) taken from the centre. A 3 mm biopsy punch was used to generate small explants (<0.5 mm^2^) of testis parenchyma from the disk periphery thus avoiding the central rete testis. Fragments of testis were transferred to a 12 well culture plate (3 pieces per well) containing DMEM (100 ul/well) supplemented with high fructose, 10% fetal bovine serum, L-glutamine (200 mM), penstrep (20 ug/ml) and gentamycin (20 ug/ml). Testis fragments were washed (Hera Cell 150i, Thermo Scientific) for 30 minutes at 37 C in 5% CO2 with gentle shaking (500 rpm–Sea Star Digital Shaker: Thomas Scientific, Swedesboro, NJ, USA) to aid diffusion of waste gases. Initially testes were incubated for 6, 12, 18 or 24 hours and processed into Bouins for histological analysis. This enabled the optimal incubation to be determined without excessive tissue necrosis. To establish optimum treatments for testosterone secretion, explants were cultured for 24 h with LH (Fisher Scientific) at 0.1, 1 and 10 ng/ml.

Testis explants from 6 dogs were washed for 30 minutes then cultured for 24 h with 0.1, 1 or10 ng/ml LH both with and without PCB153 or DEHP at 10x or 100x mean testicular concentrations. Positive control explants were incubated with dbcAMP and chemicals were made up in 0.01% DMSO (working concentration). Negative controls were incubated in the absence of LH or chemical but in the presence of 0.01% DMSO. At the end of each culture period, media was collected for testosterone analysis using the Enzo Life Sciences testosterone ELISA kit (Enzo Life Sciences, Exeter, UK); detection limit of 38.3 pg/ml; intra- and interassay cofficients of variation of 17% and 20% respectively.

### Statistical analysis

A statistical model was constructed to evaluate the change in semen quality over time whilst accounting for repeated semen measurements within dogs. Conventional multilevel models were specified that accounted for repeated correlation measures^70^ using MLwiN version 2.22^71^ with semen quality characteristics either modeled directly as a normal response variable or, when appropriate, log transformed, to ensure model residuals followed an approximately normal distribution. Possible confounding effects of the age of the dog on semen quality were tested as linear and polynomial terms. In addition confounding effects of breed (categorical variable), bodyweight (tested as linear and polynomial terms) and sire of dog (included as a random effect) were investigated. Since its inclusion had no substantive effect in any model, sire was not included as a random effect in the final models. Model specification was:


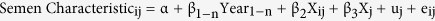


where the subscripts i, and j denote the i^th^ repeated measurement of semen quality (Percentage Normal Motility, Percentage Normal Sperm or Total Sperm Output), in the j^th^ dog, α is the regression intercept, Year_1−n_ are dummy variables representing each year of the ‘n’ study years, with β_1−n_ the related coefficients, X_ij_ are covariates associated with each semen quality reading (such as age of dog at sampling) with β_2_ the related coefficients, X_j_ are covariates associated with each dog (such as breed) with β_3_ the related coefficients, u_j_ is a random effect to account for repeated correlated measures within dogs (assumed to follow a Normal distribution with a mean of zero and variance Ω_u_) and e_ij_ is an error term reflecting residual variation between semen quality measurements (assumed to follow a Normal distribution with a mean of zero and variance Ω_e_).

Model building followed an exploratory stepwise procedure and explanatory covariates remained in the model when the significance probability was <0.05. Interaction terms between covariates were tested in the model and also remained when P < 0.05. Model fit was visually assessed using graphs of residuals at each hierarchical level^70^. The data were examined for points or higher level units with a large influence on parameter values and the models were re-run when necessary, with these points omitted, to examine their effect on parameter values.

Temporal changes in cryptorchidism, male:female ratio and mortality of male and female fetuses were assessed by linear regression analysis using GraphPad prism statistical analysis software. Chemicals (PCB153, DEHP) were tested singly and in combination as a 2 chemical by 3 concentration by 4 times split plot factorial design. Raw data collected from the Hobsons Sperm Tracker was used and comparisons made using a general ANOVA model (Genstat). Data was fitted to the model so that interactions between the concentration of each chemical and time could be assessed. Dogs were considered to be replicate blocks and sperm samples within dogs as the main plots. The main effect of concentration group was tested against the main-plot between-sample error; all other factors and their interactions were tested against the residual sub-plot error.

## Additional Information

**How to cite this article**: Lea, R. G. *et al.* Environmental chemicals impact dog semen quality in vitro and may be associated with a temporal decline in sperm motility and increased cryptorchidism. *Sci. Rep.* **6**, 31281; doi: 10.1038/srep31281 (2016).

## Supplementary Material

Supplementary Information
