# Corrigendum: Environmental chemicals impact dog semen quality in vitro and may be associated with a temporal decline in sperm motility and increased cryptorchidism

**DOI:** 10.1038/srep33267

**Published:** 2016-09-16

**Authors:** Richard G. Lea, Andrew S. Byers, Rebecca N. Sumner, Stewart M. Rhind, Zulin Zhang, Sarah L. Freeman, Rachel Moxon, Holly M. Richardson, Martin Green, Jim Craigon, Gary C. W. England

Scientific Reports 6: Article number: 31281; 10.1038/srep31281 published online: 08 09 2016; updated: 09 16 2016.

The original version of the Article contained typographical errors.

In the Abstract,

“A decline in the number of males born relative to the number of females was also observed. ECs, including diethylhexyl phthalate (DEHP) and polychlorinated bisphenol 153 (PCB153), were detected in adult dog testes and commercial dog foods at concentrations reported to perturb reproductive function in other species”.

now reads:

“A decline in the number of males born relative to the number of females was also observed. ECs, including diethylhexyl phthalate (DEHP) and polychlorinated biphenyl 153 (PCB153), were detected in adult dog testes and commercial dog foods at concentrations reported to perturb reproductive function in other species”.

In the Results section under subheading ‘Establishing concentrations of environmental chemicals in canine adult testis and semen’,

“Seven polychlorinated bisphenol (PCB) congeners, 5 polybrominated diphenyl ether (PBDE) congeners and diethylhexyl phthalate (DEHP) were detected in testis (Fig. 3a–c)”.

now reads:

“Seven polychlorinated biphenyl (PCB) congeners, 5 polybrominated diphenyl ether (PBDE) congeners and diethylhexyl phthalate (DEHP) were detected in testis (Fig. 3a–c)”.

These errors have now been corrected in the PDF and HTML versions of the Article.

