# Supplementary material for: Environmental chemicals impact dog semen quality in vitro and may be associated with a temporal decline in sperm motility and increased cryptorchidism
Source: Sci Rep. 2016 Aug 9;6:31281. doi: 10.1038/srep31281 (PMC4977511; doi:10.1038/srep31281)
Supplement: Supplementary Information [file srep31281-s1.pdf]

Supplementary Information

Environmental chemicals impact dog semen quality in vitro and may be associated with a temporal decline in sperm motility and increased cryptorchidism

Lea R.G.[1,2*], Byers A.S.[1], Sumner R.N. [1], Rhind S.M.[3↑] , Zhang Z.[3], Freeman, S.L.[1], Moxon, R.[4], Richardson H.M.[1], Green, M.[1], Craigon J.[5], England, G.C.W[1]

[1]School of Veterinary Medicine and Science, University of Nottingham, UK.

[2]School of Animal Rural and Environmental Sciences, Nottingham Trent University, UK.

[3]Environmental and Biochemical Sciences, The James Hutton Institute, UK.

[4]National Breeding Centre, Guide Dogs for the Blind Association, UK

[5]School of Biosciences, University of Nottingham, UK.

[↑] Stewart Rhind deceased March 2013

*Corresponding author: richard.lea@nottingham.ac.uk

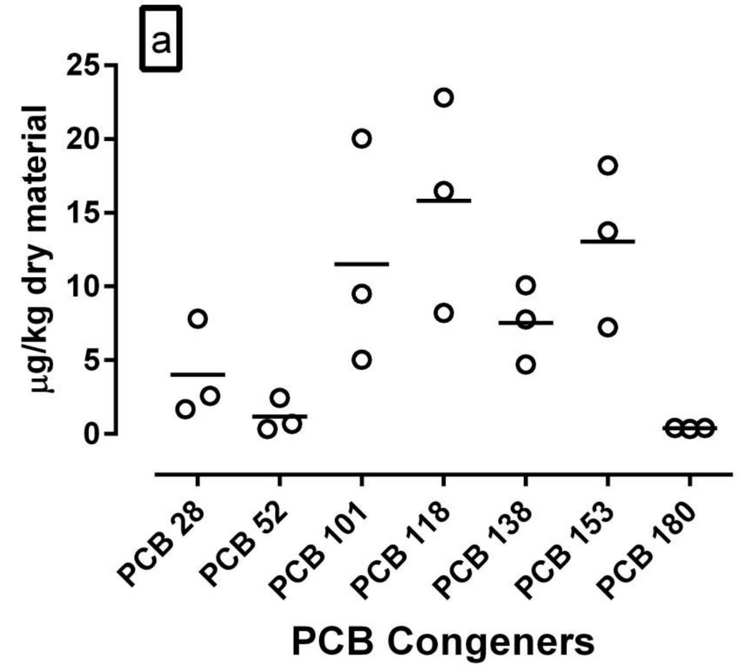

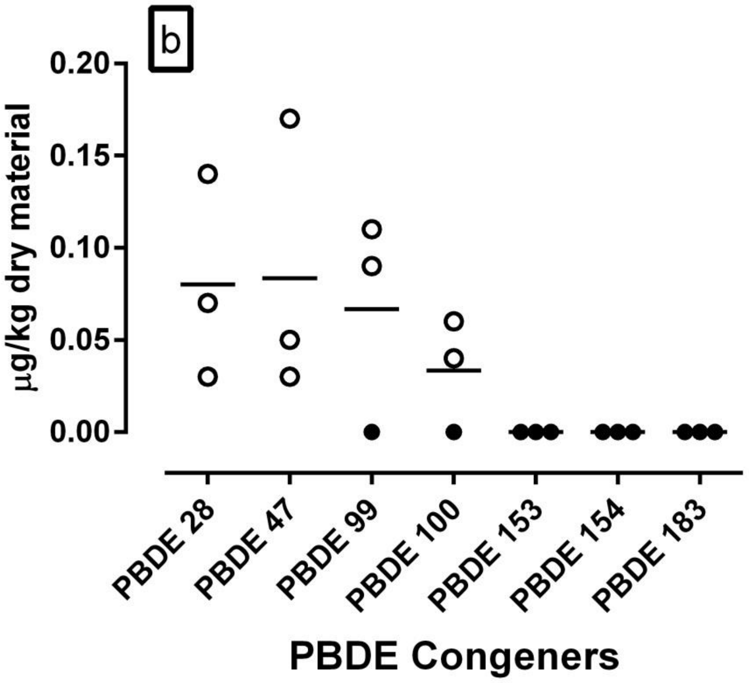

Supplementary Figure S1: Concentrations of environmental chemicals in pooled canine ejaculates. Fourteen ejaculates were collected and two pools of 4 samples and one of 5 samples generated. (a) PCB congeners, (b) PBDE congeners. Black circle = non-detected.
